# TRAF6 mediates human DNA2 polyubiquitination and nuclear localization to maintain nuclear genome integrity

**DOI:** 10.1093/nar/gkz537

**Published:** 2019-06-19

**Authors:** Yuan Meng, Changwei Liu, Lei Shen, Mian Zhou, Wenpeng Liu, Claudia Kowolik, Judith L Campbell, Li Zheng, Binghui Shen

**Affiliations:** 1 Colleges of Life Sciences, Zhejiang University, Hangzhou, Zhejiang 310027, China; 2 Department of Cancer Genetics and Epigenetics, Beckman Research Institute of City of Hope, 1500 East Duarte Road, Duarte, CA 91010, USA; 3 Department of Molecular Medicine, Beckman Research Institute of City of Hope, 1500 East Duarte Road, Duarte, CA 91010, USA; 4 Division of Chemistry and Chemical Engineering, Braun Laboratories, California Institute of Technology, Pasadena, CA 91125, USA

## Abstract

The multifunctional human DNA2 (hDNA2) nuclease/helicase is required to process DNA ends for homology-directed recombination repair (HDR) and to counteract replication stress. To participate in these processes, hDNA2 must localize to the nucleus and be recruited to the replication or repair sites. However, because hDNA2 lacks the nuclear localization signal that is found in its yeast homolog, it is unclear how its migration into the nucleus is regulated during replication or in response to DNA damage. Here, we report that the E3 ligase TRAF6 binds to and mediates the K63-linked polyubiquitination of hDNA2, increasing the stability of hDNA2 and promoting its nuclear localization. Inhibiting TRAF6-mediated polyubiquitination abolishes the nuclear localization of hDNA2, consequently impairing DNA end resection and HDR. Thus, the current study reveals a mechanism for the regulation of hDNA2 localization and establishes that TRAF6-mediated hDNA2 ubiquitination activates DNA repair pathways to maintain nuclear genome integrity.

## INTRODUCTION

DNA replication is the central process that ensures the faithful transmission of genetic information from parental cells to daughter cells (1,2). However, this process is frequently perturbed by endogenous factors and environmental agents that cause DNA damage (3–5). Replication stresses cause DNA replication forks to stall and collapse if not stabilized and repaired, resulting in mutations and chromosomal rearrangements, which are hallmarks of pre-cancerous cells (5–10). Therefore, the ability of cells to respond to DNA replication stresses is essential to maintain genomic integrity. Genetic and molecular studies indicate that the highly conserved DNA2 nuclease/helicase plays a crucial role in counteracting replication stresses (11–13). Originally identified for its role in Okazaki fragment maturation during nuclear DNA replication in yeast cells (14–20), DNA2 is now recognized as a multifunctional nuclease that is also required for double-strand break (DSB) repair (21–24) and maintaining telomere stability (25–28) in both yeast and mammalian cells. It has also been shown that yeast DNA2 (yDNA2) binds to and maintains the stability of the replication fork to prevent it from reversing and collapsing (29). yDNA2 nuclease deficiency results in large and complex DNA insertions at chromosomal breaks (30). Recently, we demonstrated that human DNA2 (hDNA2) localizes to the centromere region (31), which contains predominantly secondary structure-forming α-satellite repetitive DNA sequence (32,33). We further revealed that hDNA2 is essential for efficient replication of this region (31).

Although hDNA2 and other mammalian DNA2 proteins have enzymatic activities similar to that of yDNA2 (34–36), they lack the classic nuclear localization signal (NLS) found in yDNA2 and predominantly localize to the mitochondria under normal culture conditions (36). However, maintaining the integrity of telomeres and centromeres and processing stalled replication forks require nuclear DNA2 at secondary DNA structures, DNA lesions and stalled replication forks. This suggests that DNA2 may be transported into the nucleus in response to environmental and endogenous stimuli (37). The nuclear localization of a vast majority of nuclear proteins is mediated by the built-in NLS motif, which is a cluster of positively charged amino acid residues that binds to the nuclear transport protein importin α (38). Proteins that lack an NLS may interact with other proteins that contain an NLS to enter the nucleus via a piggyback transport mechanism (39). Several studies have also suggested that the protein ubiquitination system may mediate nuclear transport in an NLS-independent fashion (39–41).

Here, we report that nuclear hDNA2 is ubiquitinated and that hDNA2 ubiquitination is significantly increased in response to DNA-damaging agents such as camptothecin (CPT) and hydroxurea (HU). We further reveal that human TRAF6 (hTRAF6), an E3 ligase, binds to hDNA2 and mediates its K63 ubiquitination, which promotes its stability and nuclear localization. Genetic or chemical inhibition of hTRAF6 or hDNA2 activity abolishes the ubiquitination and nuclear localization of hDNA2, consequently impairing DNA end resection and homology-directed recombination repair (HDR) of DSBs. Thus, the current work reveals the role of hTRAF6-mediated ubiquitination in regulating the nuclear transport of hDNA2 to maintain nuclear genome integrity.

## MATERIALS AND METHODS

### Cell culture

All cells were cultured in Dulbecco's Modified Eagle's Medium (DMEM; Gibco) supplemented with 10% fetal bovine serum (FBS; Sigma) and 100 U/ml Penicillin/Streptomycin (Genesee Scientific). DNA2 knockdown HeLa and 293T cell lines were constructed using a lentivirus as previously described (42). Briefly, lentiviruses were produced by co-transfection of 293T cells with pResQ shDna2′, psPAX2 and pMD2.G (4:3:1 ratio). Viruses were collected 48 h post-transfection. Infection was carried out overnight in the presence of 10 μg/ml protamine sulfate. A total of 2 μg/ml of puromycin was added 48 h post-infection to select for infected cells. Knockdown efficiency was confirmed using western blot analysis. pResQ shDna2 was a gift from Sheila Stewart (Addgene plasmid #31952).

### RNA interference and drug treatment

HeLa cells were transfected with scrambled siRNA control oligos or siRNA oligos specifically against TRAF6 (Bioland) using Lipofectamine 2000 transfection reagent (Thermo Fisher) or transfected with scrambled control shRNA or shRNA pLV-EGFP:T2A:Puro-U6-hTRAF6 from VectorBuilder (https://en.vectorbuilder.com/). After 48–72 h, the cells were harvested and lysed. Knockdown efficiency was confirmed using quantitative RT-PCR. hTRAF6 siRNA targeting sequences: GCAGUGCAAUGGAAUUUAUTT, CCCAGUCACACAUGAGAAUTT and GCAAAUGUCAUCUGUGAAUTT. TRAF6 shRNA target sequence: AGCGCTGTGCAAACTATATAT.

Cycloheximide (CHX, 50 μg/ml, purity: 95%) or MG132 (1 μM, purity: >90%) was added to the cell media for 0−24 h to inhibit protein synthesis or degradation, respectively. CPT (1 μM; purity: 99%; Sigma) or HU (1 mM; purity: 98%; Sigma) was added to the cell media for 4 or 16 h, respectively, before cell collection. The TRAF6 E3 ligase inhibitor C25–140 (ChemDiv, Catalog #G827–0140) was added for 48 h at a final concentration of 30 μM. The TRAF6 inhibitory peptide T6PD (Novus Biologicals, Catalog #NBP2–26506) was added for 24 h at a final concentration of 100 μM. The ataxia-telangiectasia mutated (ATM) inhibitor KU-55933 (Abcam, Catalog #ab120637) was added for 12 h at a final concentration of 10 μM.

### Protein expression and purification

Recombinant protein expression in mammalian cells and protein purification were conducted as previously described (27). Briefly, 293T cells were transfected with 3xFlag-hDNA2 or myc-hTRAF6 using the Polyjet (SignaGen) transfection reagent. Twenty-four hours after transfection, the cells were collected and lysed by brief sonication in the immunoprecipitation (IP) buffer H150 (50 mM HEPES-KOH [pH7.4], 150 mM NaCl, 0.1% NP40 and 10% glycerol) with protein inhibitor cocktail (Thermo Fisher). After centrifugation (20,000 × *g*, 15 min, 4°C), the supernatant was incubated with anti-Flag M2 magnetic beads (Sigma) or anti-myc magnetic beads (Thermo Fisher) overnight. The beads were washed with H500 buffer (50 mM HEPES-KOH [pH7.4], 500 mM NaCl, 0.1% NP40 and 10% glycerol) and H150 buffer (50 mM HEPES-KOH [pH7.4], 150 mM NaCl, 0.1% NP40 and 10% glycerol) once, then eluted with 250 μg/ml 3xFlag peptide or myc peptide (Apex) for 6 h.

### Immunoprecipitation

293T cells were transfected with 3xFlag-hDNA2 or myc-hTRAF6 alone or in combination using the Polyjet (SignaGen) transfection reagent. Twenty-four hours after transfection, the cells were collected and lysed by brief sonication in the IP buffer H150 with protein inhibitor cocktail (Thermo Fisher). After centrifugation (20,000 × *g*, 15 min, 4°C), the supernatant was incubated with anti-Flag M2 magnetic beads (Sigma) or anti-myc magnetic beads (Thermo Fisher) overnight. The beads were washed three times with H150 buffer, and then eluted with 250 μg/ml 3xFlag peptide or myc peptide (Apex) for 6 h. The 3xFlag-hDNA2 or myc-hTRAF6 was subjected for analysis by western blot analysis or mass spectrometry.

To immunoprecipitate endogenous hDNA2, HeLa cell lysate was incubated with an anti-hDNA2 antibody and Protein A/G dynabeads in the IP buffer overnight. The beads were washed three times with H500 buffer and subjected to boiling in 1× sodium dodecyl sulphate-polyacrylamide gelelectrophoresis (SDS-PAGE) loading buffer directly. hDNA2 levels were analyzed by western blot analysis.

### Mass spectrometry

To detect the DNA2 ubiquitination sites, 3xFlag-hDNA2, myc-hTRAF6 and 6His-tagged ubiquitin were co-transfected in 293T cells. Total 3xFlag-hDNA2 proteins were purified using anti-Flag M2 beads. Purified 3xFlag-hDNA2 proteins were resolved in an 8% SDS-PAGE gel. The gel was stained with Coomassie, and the DNA2 bands were cut and subjected to mass spectrometry (Shanghai Institute of Material Medica, Chinese Academy of Sciences). Briefly, the excised gel containing 3xFlag-hDNA2 was cut into pieces and washed with water. It was then destained and dehydrated with acetonitrile. Dithiothreitol (DTT) and NH_4_HCO_3_ were added to final concentrations of 20 mM and 50 mM, respectively. The mixture was incubated at 56°C for 30 min. The gel was destained with acetonitrile again. Iodoacetamide (IAA) and NH_4_HCO_3_ were added to final concentrations of 100 mM and 50 mM, respectively. After incubation for 20 min in the dark, in-gel tryptic digestion was carried out at 37°C overnight. The digested sample was dissolved in 0.1% formic acid and cleared by centrifugation (room temperature, 20,000 × *g*, 15 min). The supernatant was analyzed using an Orbitrap Elite Hybrid Mass Spectrometer (Thermo Fisher). The data was analyzed using MaxQuant software (43).

### Subcellular fractionation

Subcellular fractionation of proteins into cytoplasmic and nuclear fractions was performed according to the previously published protocol with slight modifications (44). Briefly, harvested HeLa or 293T cells were washed with hypotonic buffer (20 mM Tris–HCl [pH7.5], 10 mM NaCl, 10% glycerol, 1 mM phenylmethylsulfonyl fluoride (PMSF) and protein inhibitor cocktail) without detergent. The cells were then incubated in hypotonic buffer containing 0.1% NP40 on ice for 5 min. The cell lysate was centrifuged at low speed (1,300 × *g*, 5 min, 4°C). The supernatant was transferred into a new 1.5 ml tube and subjected to another round of centrifugation (20,000 x *g*, 15 min, 4°C). The cleared supernatant was kept as the cytoplasmic fraction. The nuclear fraction was extracted using nuclear extraction buffer (20 mM Tris–HCl [pH7.5], 500 mM NaCl, 10% glycerol, 0.1% NP40, 1 mM PMSF and protein inhibitor cocktail). After brief sonication, the pellets were centrifuged (20,000 × *g*, 15 min, 4°C). The supernatant was kept as the nuclear fraction.

Subcellular fractionation of proteins into cytosolic, mitochondrial and nuclear fractions was performed using the Mitochondrial Isolation Kit for Cultured Cells (Thermo Scientific) using the protocol provided by the supplier with modifications. Briefly, 20 million cells expressing 3xFlag-hDNA2 or 3xFlag-hDNA2 and myc-hTRAF6 were washed with phosphate buffered saline (PBS) and incubated on ice in Reagent A for 2 min. Reagent B was added, and the mixture was incubated on ice for 5 min. Reagent C was added, and the contents were mixed by inverting the tube several times, followed by centrifugation (700 × *g*, 10 min, 4°C). The supernatant (cytosol plus mitochondria) was collected and centrifuged (12,000 ×*g*, 15 min, 4°C). The supernatant (cytosol) was centrifuged (20,000 × *g*, 15 min, 4°C) to remove trace amounts of debris. The pellet (mitochondria) was washed with Reagent C twice to remove cytosolic contamination. The nuclear pellet was washed twice with wash buffer (10 mM Tris–HCl [pH 7.5], 10 mM KCl and 0.4% NP) to remove non-lysed cells and mitochondrial contaminants. A total of 1 mM PMSF and protease inhibitor cocktail were added to all solutions before use.

### *In vitro* ubiquitination assays

*In vitro* ubiquitination assays were performed, as previously described (42), using the Ubiquitin Conjugation Kit (Boston Biochem) according the manufacturer's instructions. Briefly, 100 ng 3xFlag-hDNA2 and the E2 mixture from the kit were reacted either with the E3 mixture from the kit or an equivalent amount of purified myc-hTRAF6 at 37°C in a water bath for 90 min. Reactions were detected by western blot analysis using an anti-ubiquitin antibody.

### Immunofluorescence

Detection of phosphor-RPA (S33) was carried out following the previously published protocol (11). Briefly, HeLa cells were grown on coverslips. After transfection and/or drug treatment, the cells were fixed with 4% paraformaldehyde (PFA) at room temperature for 30 min and permeabilized in 0.15% Triton X-100 for 15 min at room temperature. The coverslips were blocked with 5% bovine serum albumin for 1 h and then incubated with the primary antibody (specified in the figure legends) at room temperature for 2 h or at 4°C overnight. After washing with PBS buffer, the coverslips were incubated with Alexa-555-conjugated secondary antibodies (1:150, Life Technologies) at room temperature for 1 h. After washing with PBS, the coverslips were incubated with DAPI solution (Thermo Fisher, Catalog #62248) to stain the nuclei. The coverslips were mounted with ProLong™ Gold Antifade Mountant (Thermo Fisher, Catalog #P36930) and examined by fluorescence microscopy.

### DR-GFP-based HDR assay

293T cells carrying the DR-GFP reporter construct were generated according to a previously published protocol (45). DNA fragments encoding a pair of single-stranded guide RNAs (sgRNAs) and Cas9 D10A nickase were subcloned into the multiplex CRISPR/Cas9 vector to simultaneously express the sgRNAs and Cas9 D10A (46). The HDR of CRISPR/Cas9 D10A-induced DSBs in the DR-GFP reporter construct was carried out as described (45). Briefly, 3 × 10^5^ cells were seeded. Following pre-treatment with DMSO, C5 or C25–140, the cells were transfected with the multiplex gRNA/CAS9 D10A vector targeting the DR-GFP reporter (0.4 μg) using Lipofectamine 2000 transfection mixes. The percentage of GFP+ cells was determined using fluorescence-activated cell sorting (FACS) analysis 3 days after transfection.

### Quantification and statistical analyses

The intensity of immunofluorescence staining was quantified using ImageJ software (NIH). All data are presented as the mean ± SEM of at least three independent experiments. *P*-values for differences between groups were calculated using two-tailed Student's *t*-tests. *P* < 0.05 was considered statistically significant.

## RESULTS

### Nuclear hDNA2 is ubiquitinated

**Figure 1.**
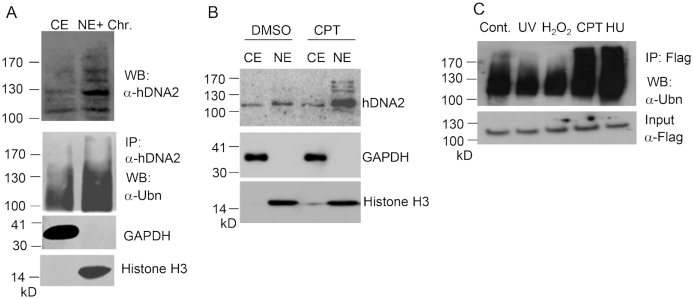
Nuclear hDNA2 is ubiquitinated. (**A**) Immunoprecipitation and western blot analysis of hDNA in cytosplasmic extract (CE) and NE and soluble chromatin (chr) fractions of HeLa cells using anti-hDNA2 or anti-ubiquitin (Ubn) antibodies. GAPDH and histone H3 were used as CE and NE+Chr markers, respectively. (**B**) HeLa cells were treated with DMSO (control) or CPT (1 μM). After 4 h, hDNA2 in the CE and NE was analyzed by western blot analysis using an anti-hDNA2 antibody. GAPDH and histone H3 were used as CE and NE markers, respectively. (**C**) 293T cells overexpressing 3xFlag-hDNA2 were treated with UV (250 J/m^2^), H_2_O_2_ (1 mM, 15 min), CPT (1 μM, 4 h) or HU (1 mM, 16 h). 3xFlag-hDNA2 was purified using anti-Flag M2 beads and its ubiquitination status was evaluated by western blot analysis using an anti-ubiquitin antibody.

To test if ubiquitination or other post-translational modifications of hDNA2 are required for its nuclear localization, we isolated cytoplasmic extracts (CE) and nuclear extracts (NE) from HeLa cells and analyzed hDNA2 proteins using western blot analysis. Cytoplasmic hDNA2 had one major band corresponding to the molecular weight of non-modified hDNA2 (∼110 kD) and faint bands with molecular weights >110 kD. In contrast, nuclear hDNA2 showed distinct bands with molecular weights greater than 120 kD (Figure 1A). We observed that, in response to treatment with the DNA-damaging and fork-stalling agent CPT, the amount of total hDNA2, as well as nuclear hDNA2, increased considerably (Figure 1B). Importantly, levels of nuclear hDNA2 molecules with molecular weights higher than 120 kD increased, indicating that covalent attachments of ubiquitin or SUMO peptides to the hDNA2 protein may be important for its stability and nuclear translocation. We then used an anti-hDNA2 antibody to pull down endogenous hDNA2 from the CE and NE. Western blot analysis of the immunoprecipitated cytoplasmic and nuclear hDNA2 revealed that nuclear hDNA2 was ubiquitinated (Figure 1A) but not SUMOylated (data not shown). By overexpressing 3xFlag-tagged hDNA2 (3xFlag-hDNA2) in 293T cells, we further confirmed that nuclear hDNA2 was ubiquitinated (Figure 1C). Moreover, upon treatment of the cells with CPT or HU, hDNA2 ubiquitination increased substantially (Figure 1C). These findings suggest that, in response to replication stress induced by environmental agents, hDNA2 is ubiquitinated and subsequently translocated into the nucleus.

### hTRAF6 interacts with hDNA2 and catalyzes its ubiquitination

**Figure 2.**
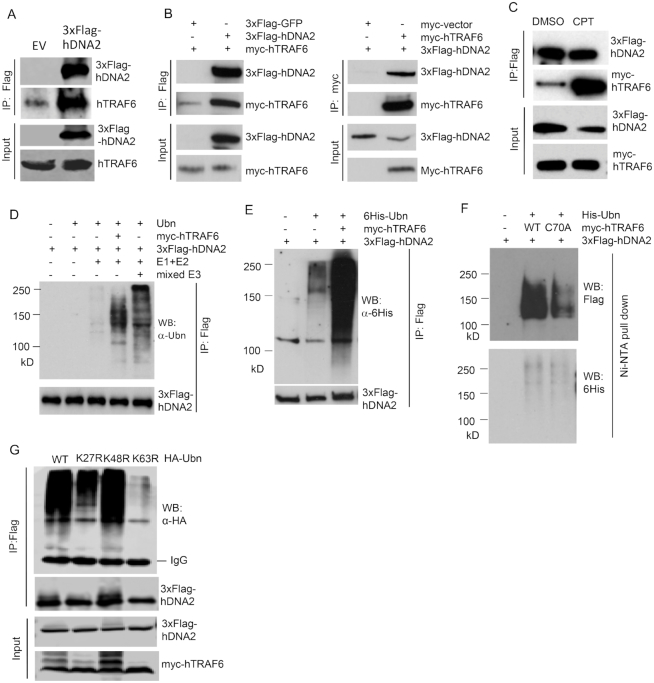
hTRAF6 interacts with hDNA2 and catalyzes its ubiquitination. (**A**) 293T cells were transfected with a 3xFlag-hDNA2 or an empty control vector (EV). The 3xFlag-hDNA2 complex was pulled down using anti-Flag M2 beads. The endogenous hTRAF6 in complex with 3xFlag-hDNA2 was detected using anti-hTRAF6 antibody. (**B**) 3xFlag-hDNA2 and myc-hTRAF6 were co-expressed in 293T cells. In control reactions, myc-hTRAF6 was co-expressed with 3xFlag-GFP and 3xFlag-hDNA2 was co-expressed with a non-specific peptide encoded by the empty myc vector. 3xFlag-hDNA2 and the myc-hTRAF6 complex were pulled down using anti-Flag M2 or anti-myc affinity beads. myc-hTRAF6 and 3xFlag-hDNA2 were detected by western blot analysis using anti-myc or anti-Flag antibodies. (**C**) 293T cells co-expressing 3xFlag-hDNA2 and myc-hTRAF6 were treated with DMSO or CPT (1 μM, 4 h). 3xFlag-hDNA2 and myc-hTRAF6 were pulled down and analyzed by western blot, as described for panel (B). (**D**) *In vitro* reconstitution of hDNA2 ubiquitination using ubiquitin (Ubn) and purified recombinant myc-hTRAF6, 3xFlag-hDNA2, ubiquitination enzymes E1 and E2, and mixed E3 ligases. (**E**) 3xFlag-hDNA2 was co-expressed with 6His-tagged Ubn (6His-Ubn) and myc-hTRAF6 in 293T cells. Total hDNA2 was isolated using anti-Flag M2 beads and the ubiquitination status of 3xFlag-hDNA2 was analyzed by western blot analysis using an anti-6His antibody. **(F)** 3xFlag-hDNA2 was co-expressed with 6His-Ubn and WT myc-hTRAF6 or C70A (ubiquitin E3 ligase activity-deficient) mutant myc-hTRAF6 in 293T cells. Total ubiquitinated proteins were isolated using Ni-NTA beads under denaturing conditions (8 M urea). 6His-Ubn-linked hDNA2 was detected by western blot analysis using an anti-Flag antibody. (**G**) 3xFlag-hDNA2 was co-expressed with HA-tagged WT, K27R, K48R or K63R ubiquitin (HA-Ubn) and myc-hTRAF6 in 293T cells. Total hDNA2 was isolated using anti-Flag M2 beads, and the ubiquitination status of 3xFlag-hDNA2 was analyzed by western blot analysis using an anti-HA antibody.

We subsequently sought to identify the ubiquitin E3 ligase that ubiquitinates hDNA2 and mediates hDNA2 nuclear translocation in response to DNA-damaging and fork-stalling agents. A previous study revealed that ATM kinase, the master DNA damage-sensing protein (47), migrates to the cytoplasm in response to DNA damage (48). Cytoplamic ATM then activates the ubiquitin E3 ligase TRAF6, which catalyzes K63-linked polyubiquitination (49), to transduce the DNA damage signal to the NF-κB pathway to regulate cell proliferation and survival (48). It is possible that cytoplasmic ATM-triggered hTRAF6 activation also mediates the nuclear translocation of DNA repair proteins, such as hDNA2, in response to DNA damage. To determine if hTRAF6 mediates hDNA2 nuclear localization, we first expressed 3xFlag-hDNA2 in 293T cells and conducted co-immunoprecipitation assays to determine if hDNA2 and hTRAF6 interact. We found that endogenous hTRAF6 was pulled down with 3xFlag-hDNA2 (Figure 2A). To further verify that hDNA2 interacts with hTRAF6, we co-expressed 3xFlag-hDNA2 and myc-tagged hTRAF6 (myc-hTRAF6) in 293T cells and conducted co-immunoprecipitation assays. We found that myc-hTRAF6 was pulled down with 3xFlag-hDNA2 but not 3xFlag-GFP using anti-Flag M2 beads (Figure 2B). Consistent with this finding, 3xFlag-hDNA2 was pulled down with myc-hTRAF6 using anti-myc beads (Figure 2B). Furthermore, we found that CPT treatment of cells co-expressing 3xFlag-hDNA2 and myc-hTRAF6 stimulated the binding of myc-hTRAF6 and 3xFlag-hDNA2 (Figure 2C).

To determine if hTRAF6 ubiquitinates hDNA2, we reconstituted hDNA2 ubiquitination reactions using the ubiquitination enzymes E1 and E2 with or without purified myc-hTRAF6 or a mixture of other ubiquitin E3 ligases. In the absence of a ubiquitin E3 ligase, little hDNA2 was ubiquitinated (Figure 2D). On the other hand, the addition of purified recombinant hTRAF6 greatly enhanced the ubiquitination of hDNA2 (Figure 2D). hDNA2 was also ubiqutinated with the addition of a mixture of E3 ligases (Figure 2D), suggesting that E3 ligases other than hTRAF6 may also catalyze hDNA2 ubiquitination *in vitro*. Co-immunoprecipitation using anti-Flag M2 beads and western blot analysis indicated that overexpression of myc-hTRAF6 enhanced the ubiquitination of 3xFlag-hDNA2 (Figure 2E). To further demonstrate that hTRAF6 mediates hDNA2 ubiquitination, we co-expressed 3xFlag-hDNA2, 6His-tagged ubiquitin and wild-type (WT) hTRAF6 or the E3 ligase activity-deficient hTRAF6 mutant C70A in 293T cells. We isolated 6His-tagged ubiquitin-linked proteins under denaturing conditions (8 M urea), and western blot analysis of the 6His-tagged ubiquitin-linked hDNA2 revealed that the expression of hTRAF6 enhanced hDNA2 ubiquitination; however, this effect was dramatically lower for the C70A hTRAF6 mutant compared to WT hTRAF6 (Figure 2F). It is worth noting that expression of C70A hTRAF6 did not completely abolish ubiquitination of hDNA2 (Figure 2F). This suggests that there are other E3 ligases that mediate different forms of hDNA2 polyubiquitination in these cells. In addition, we tested the effects of K27R, K48R and K63R ubiquitin mutations on hDNA2 ubiquitination. We found that considerably less K27R and K63R ubiquitin was linked to hDNA2 than WT and K48R ubiquitin in 293T cells expressing exogenous hTRAF6 (Figure 2G). This finding is consistent with findings that TRAF6 mediates K27-linked (50) and K63-linked ubiquitination (49).

### hTRAF6-mediated ubiquitination enhances hDNA2 stability and nuclear localization

**Figure 3.**
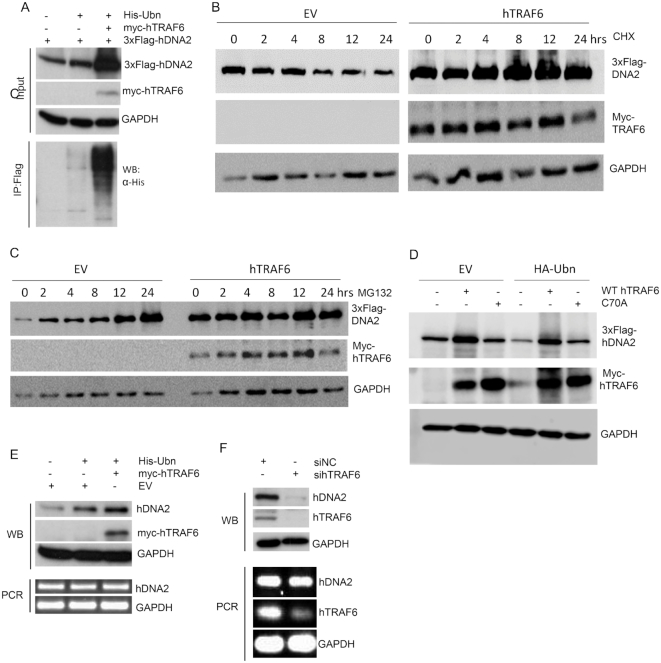
hTRAF6-mediated hDNA2 ubiquitation is important for the stability of hDNA2. (**A**) 3xFlag-hDNA2 was expressed alone or with 6His-Ubn and/or myc-hTRAF6 in 293T cells. 3xFlag-hDNA2 was detected by western blot analysis using an anti-Flag antibody. GAPDH was used as an internal control. The ubiquitination status of 3xFlag-hDNA2 was analyzed as described for Figure 2D. (**B** and **C**) 293T cells were transfected with 3xFlag-hDNA2 and an empty control vector (EV) or myc-hTRAF6. The cells were treated with CHX (panel B, 50 μg/ml) or MG132 (Panel C, 1 μM) and harvested and lysed at 0, 2, 4, 8, 12 or 24 h. 3xFlag-hDNA2 levels were determined by western blot using anti-Flag antibody. GAPDH was used as a loading control. (**D**) 3xFlag-hDNA2 was co-expressed with WT or C70A mutant myc-hTRAF6 and with empty vector (EV) or the vector expressing HA-ubiquitin (HA-Ubn). In the controls, 3xFlag-hDNA2 expression vector was co-transfected with EV or HA-Ubn vector. 3xFlag-hDNA2 levels were determined by western blot using anti-Flag antibody. (**E**) 6His-Ubn and/or myc-hTRAF6 were overexpressed in HeLa cells, and endogenous hDNA2 protein and mRNA levels were evaluated by western blot analysis and RT-PCR, respectively. HeLa cells harboring EV only were used as the control. GAPDH was used as an internal control for comparison of hDNA2 levels in different cells. (**F**) HeLa cells were transfected with a scrambled negative control siRNA (siNC) or siRNA against hTRAF6 (sihTRAF6). Endogenous hDNA2 protein and mRNA levels were evaluated by western blot analysis and RT-PCR, respectively. hTRAF6 knockdown efficacy was confirmed by RT-PCR. GAPDH was used as an internal control.

We sought to determine if hTRAF6 plays a role in modulating hDNA2 protein levels and nuclear translocation. We first evaluated hDNA2 protein and mRNA levels in cells with hTRAF6 overexpressed or knocked down. Western blot analysis revealed that overexpression of ubiquitin and myc-hTRAF6 greatly enhanced 3xFlag-hDNA2 ubiquitination, as well as total 3xFlag-hDNA2, in 293T cells (Figure 3A). To further assess the impact of hTRAF6 on hDNA2 stability, we treated cells expressing 3x-Flag-hDNA2 or 3xFlag-hDNA2 and myc-hTRAF6 with cycloheximide (CHX) to block protein synthesis or with MG132 to block proteasome-mediated protein degradation. We observed that 3xFlag-hDNA2 levels were gradually reduced with prolonged CHX treatment in cells without hTRAF6, but expression of hTRAF6 prevented such reduction (Figure 3B). Meanwhile, 3xFlag-hDNA2 gradually accumulated upon MG132 treatment in cells without hTRAF6 and was at consistently high levels, even without MG132 treatment, in cells with hTRAF6 (Figure 3C), suggesting that expression of hTRAF6 blocks proteasome-mediated hDNA2 degradation. To determine if TRAF6-mediated hDNA2 ubiquitination is important for hDNA2 stabilization, we co-expressed 3xFlag-hDNA2 with WT or C70A hTRAF6 and with or without HA-tagged ubiquitin. We observed that WT hTRAF6 induced hDNA2 ubiquitination and stability, but the catalysis-defective C70A mutant only partially enhanced hDNA2 stabilization (Figure 3D). These results suggest that hTRAF6-mediated ubiquitination of hDNA2 increases its stability, likely by blocking the proteasome-mediated degradation of hDNA2. In addition, the physical interaction between hTRAF6 and hDNA2 may also contribute to preventing hDNA2 degradation. Consistent with these findings, overexpression of ubiquitin and myc-hTRAF6 increased endogenous hDNA2 expression in HeLa cells (Figure 3E). Conversely, knockdown of hTRAF6 diminished endogenous hDNA2 in HeLa cells (Figure 3F). However, the overexpression and knockdown of hTRAF6 did not change hDNA2 mRNA levels (Figure 3E and F). These findings suggest that hDNA2 ubiquitination by hTRAF6 enhances its stability in a post-translational manner.

**Figure 4.**
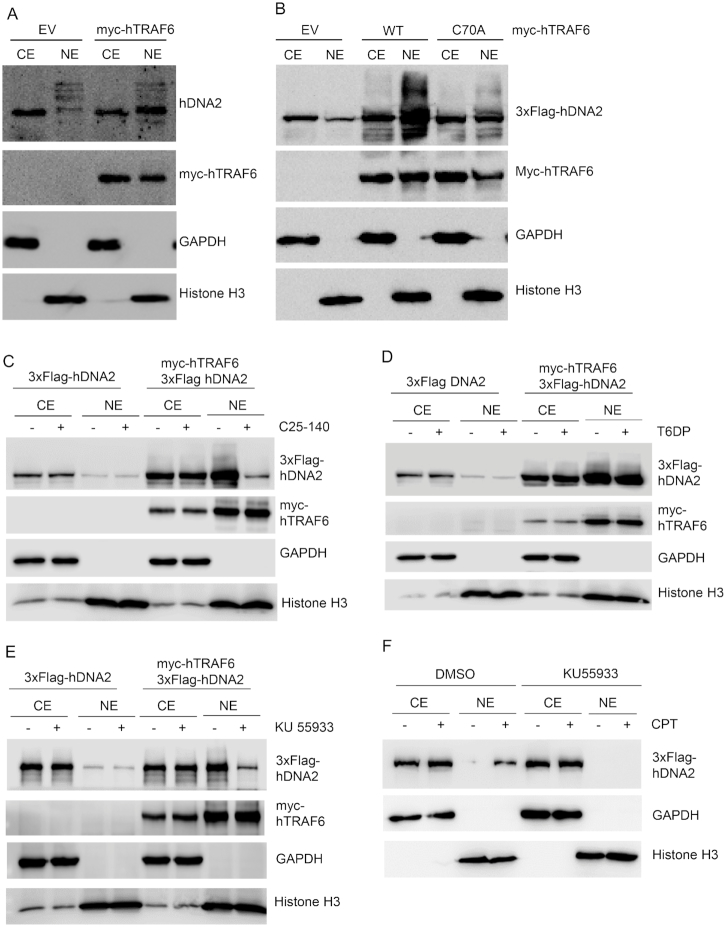
hTRAF6 promotes hDNA2 nuclear localization. (**A**) HeLa cells were transfected with an empty control vector (EV) or a vector encoding myc-hTRAF6. Endogenous hDNA2 protein in the CE and NE was analyzed by western blot analysis using an anti-hDNA2 antibody. GAPDH and histone H3 were used as CE and NE markers, respectively. (**B**) 3xFlag-hDNA2 was co-expressed with EV or a vector encoding WT or C70A (ubiquitin E3 ligase activity-deficient) mutant hTRAF6 in 293T cells. 3xFlag-hDNA2 in the CE and NE was analyzed by western blot analysis using an anti-Flag antibody. GAPDH and histone H3 were used as CE and NE markers, respectively. (**C**–**E**) 293T cells co-expressing 3xFlag-hDNA2 and myc-hTRAF6 were left untreated or were treated with (**C**) a small molecule inhibitor of TRAF6 E3 ligase activity (C25–140, 30 μM, 48 h), (**D**) a TRAF6/RANK interaction-disrupting peptide (T6DP, 100 μM, 24 h) or (**E**) a small molecule ATM kinase activity inhibitor (KU-55933, 10 μM, 16 h). 3xFlag-hDNA2 levels in the CE and NE were analyzed by western blot analysis using an anti-Flag antibody. GAPDH and histone H3 were used as CE and NE markers, respectively. (**F**) 293T cells expressing 3xFlag-hDNA2 were pre-treated with DMSO (control) or KU-55933 (10 μM, 16 h). The cells were left untreated or were treated with CPT (1 μM, 4 h). 3xFlag-hDNA2 levels in the CE and NE were analyzed by western blot analysis using an anti-Flag antibody. GAPDH and histone H3 were used as CE and NE markers, respectively.

We then determined if hDNA2 nuclear localization was dependent on hTRAF6-mediated ubiquitination of hDNA2. We conducted western blot analysis of hDNA2 in the CE and NE from cells with or without hTRAF6 overexpression. We found that overexpression of hTRAF6 promoted nuclear localization of endogenous hDNA2 (Figure 4A). To further investigate the impact of hTRAF6 on hDNA2 ubiquitination and nuclear localization, we overexpressed 3xFlag-hDNA2 with or without co-expression of myc-hTRAF6. Western blot analysis revealed that overexpression of WT, but not C70A mutant hTRAF6, greatly induced ubiquitination of 3xFlag-hDNA2 in both CE and NE and enhanced nuclear localization of 3xFlag-hDNA2 (Figure 4B). Furthermore, in the presence of C25–140, a small molecule that specifically inhibits hTRAF6 ubiquitin E3 ligase activity (51), hDNA2 ubiquitination and nuclear localization were greatly inhibited (Figure 4C). In contrast, a cell-permeable TRAF6 decoy peptide (T6DP), which specifically disrupts the TRAF6/RANK interaction (52), had little impact on TRAF6-induced hDNA2 ubiquitination and nuclear localization (Figure 4D). We also noticed that in the presence of C25–140, TRAF6 could at least partially stabilize hDNA2 (Figure 4C). This observation is consistent with our suggestion that the physical interaction between TRAF6 and hDNA2 contributes to the prevention of hDNA2 degradation. In addition, we found that the ATM inhibitor KU55933 (53) also inhibited hTRAF6-mediated hDNA2 nuclear localization (Figure 4E). This is consistent with previous findings that hTRAF6 activation in response to DNA damage is mediated by ATM (48). To determine if treatment with CPT induces hDNA2 nuclear localization in an ATM-dependent manner, we treated 293T cells expressing 3xFlag-hDNA2 with CPT in the presence or absence of KU55933. We found that KU55933 blocked CPT-induced hDNA2 nuclear localization (Figure 4F).

### Ubiquitination at the N-terminus of hDNA2 is critical for its stability and nuclear localization

**Figure 5.**
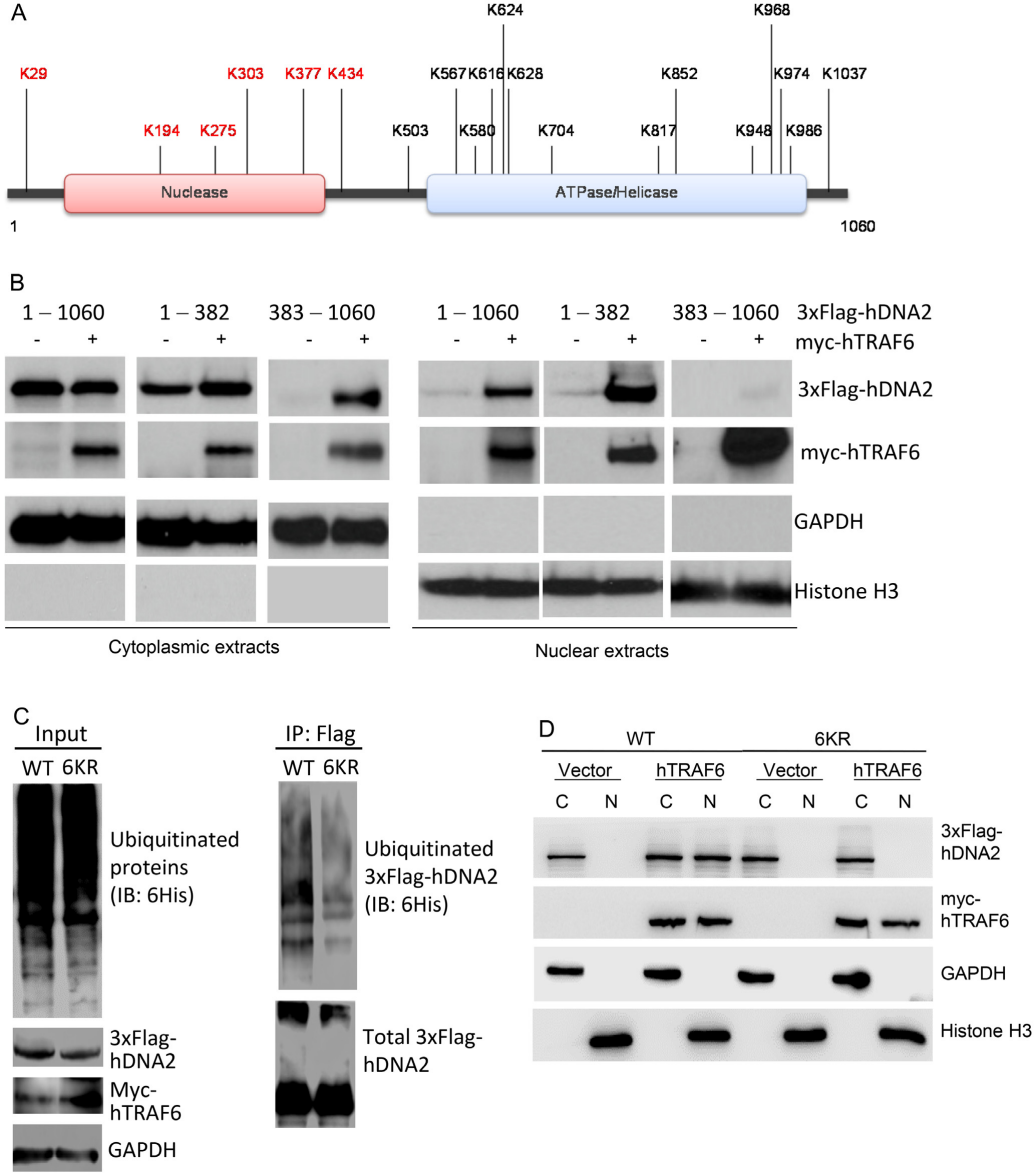
Ubiquitination at the N-terminal nuclease domain of hDNA2 is critical for its stability and nuclear localization (See also Supplementary Figures S1 and 2). (**A**) The domain structure and ubiquitinated lysine residues of hDNA2 that were identified by mass spectrometry. (**B**) 3xFlag-hDNA2 protein (full-length [1–1060], N-terminal [1–382], or C-terminal [383–1060]) was overexpressed alone or with myc-hTRAF6 in 293T cells. 3xFlag-hDNA2 protein levels in the CE and NE were analyzed by western blot analysis using an anti-Flag antibody. GAPDH and histone H3 were used as markers for the cytoplasmic and nuclear fractions, respectively. (**C**) Immunoprecipitation and western blot analysis of hTRAF6-mediated ubiquitination of WT or 6KR mutant hDNA2, conducted as described for Figure 2D. (**D**) The subcellular localization of 3xFlag WT and 6KR mutant hDNA2 in the cytoplasmic (C) and nuclear (N) fractions was analyzed as described for panel (**B**).

We used mass spectrometry to identify the hTRAF6-mediated ubiquitination sites in ubiquitinated hDNA2 isolated from 293T cells co-expressing 3xFlag-hDNA2, myc-hTRAF6 and ubiquitin. A total of 20 lysine residues were identified (Figure 5A and Supplementary Figure S1). To determine which sites are important for the nuclear localization of hDNA2, we expressed and purified the N-terminal nuclease domain (amino acids 1–382) and C-terminal ATPase and helicase domain (amino acids 383–1060) of 3xFlag-hDNA2. We overexpressed full-length, N-terminal and C-terminal 3xFlag-hDNA2 in 293T cells alone or with myc-hTRAF6. Without hTRAF6 overexpression, C-terminal hDNA2 was undetectable, compared to full-length and N-terminal hDNA2, in CE (Figure 5B). With hTRAF6 overexpression, C-terminal hDNA2 was detected, albeit at much lower levels than full-length and N-terminal hDNA2. This observation is consistent with our finding that hTRAF6 promotes hDNA2 stability and suggests that hTRAF6-mediated stability of hDNA2 occurs predominantly through its N-terminal ubiquitination, although C-terminal ubiquitination can also stabilize hDNA2 in the absence of the N-terminal if TRAF6 is overexpressed. Furthermore, overexpression of hTRAF6 stimulated the nuclear localization of N-terminal hDNA2 but not C-terminal hDNA2 (Figure 5B), suggesting that the ubiquitination sites in the N-terminus of hDNA2 play a predominant role in hTRAF6-mediated hDNA2 nuclear translocation. We therefore constructed a mutant by simultaneous substituting the N-terminal ubiquitination site lysine residues (K29, K194, K275, K303, K377 and K434) with arginine residues (6KR). The 6KR mutant hDNA2 had remarkably reduced ubiquitination compared to WT hDNA2 (Figure 5C) and nearly failed to translocate into the nucleus (Figure 5D). Individual substitution of K29, K194, K275, K303 and K434 with the arginine residue resulted in only partial defects in hTRAF6-mediated nuclear localization of hDNA2 (Supplementary Figure S2).

**Figure 6.**
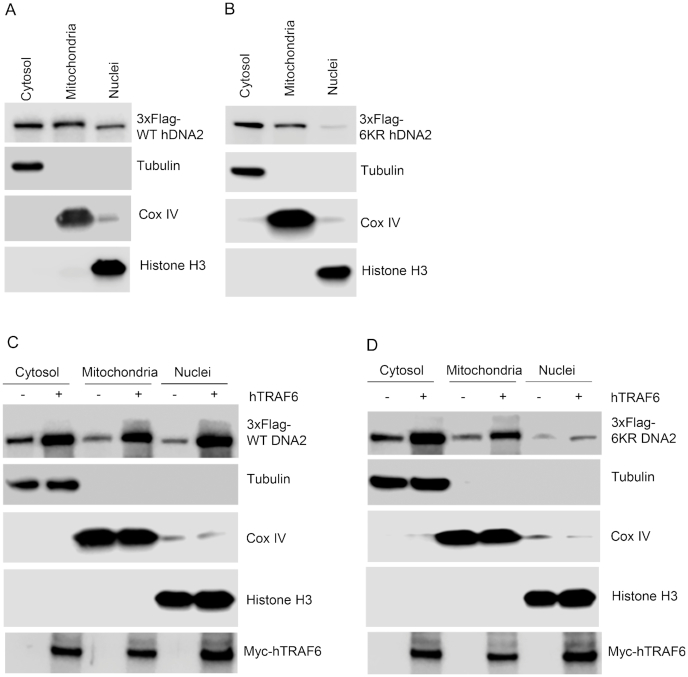
hTRAF6-mediated ubiquitination of hDNA2 enhances its nuclear and mitochondrial localization. (**A** and **B**) Subcellular localization of (A) WT and (B) 6KR 3xFlag-hDNA2 in fractionated 293T cells. Tubulin, Cox IV and histone H3 were used as markers of the cytosolic, mitochondrial and nuclear fractions, respectively. (**C** and **D**) Subcellular localization of (C) WT and (D) 6KR 3xFlag-hDNA2 in the presence or absence of myc-hTRAF6 expression in fractionated 293T cells. Tubulin, Cox IV and histone H3 were used as markers of the cytosolic, mitochondrial and nuclear fractions, respectively.

Next, we investigated if TRAF6-mediated hDNA2 ubiquitination affected its mitochondrial localization. We confirmed that 3xFlag-hDNA2, similar to endogenous hDNA2, localized to mitochondria and nuclei (Figure 6A). On the other hand, although the 6KR mutant hDNA2 also localized into mitochondria, only a little 6KR mutant protein localized to the nuclei (Figure 6B). When we co-expressed WT and 6KR hDNA2 with myc-hTRAF6 in 293T cells, we found that expression of myc-hTRAF6 enhanced both the mitochondrial and nuclear localization of WT hDNA2 (Figure 6C). In contrast, although expression of myc-hTRAF6 considerably increased the mitochondrial localization of 6KR, it did not affect the nuclear localization of 6KR (Figure 6D). These findings indicate that hTRAF6-mediated ubiquitination regulates the mitochondrial and nuclear localization of hDNA2, and ubiquitination at its N-terminus is specifically crucial for its nuclear localization.

### hTRAF6-mediated hDNA2 ubiquitination facilitates DNA end resection and HDR

**Figure 7.**
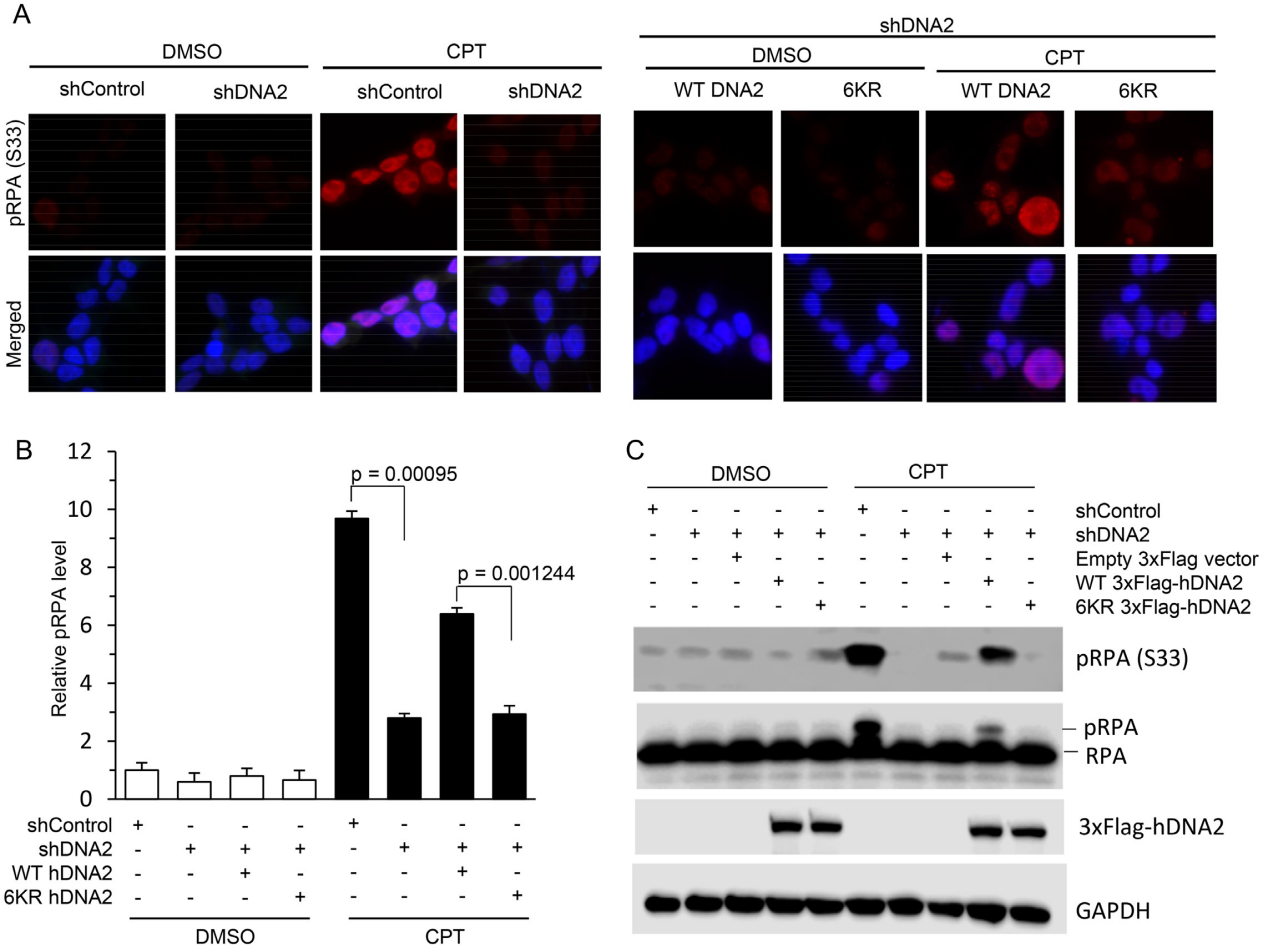
The hDNA2 6KR mutation impairs CPT-induced ssDNA production. Staining for pRPA was conducted using an anti-pRPA (S33) antibody to detect CPT-induced ssDNA regions, which is a readout of CPT-induced fork processing. (**A**) Representative immunofluorescence images for pPRA in HeLa cells transfected with scrambled control shRNA (shControl) or shRNA against hDNA2 (shDNA2), plus plasmids expressing WT or 6KR hDNA2, upon treatment with DMSO or CPT (1 μM, 4 h). Green: 3xFlag-hDNA2; red: pRPA (s33); blue: DAPI-stained nuclei. (**B**) Quantification of pRPA in HeLa cells under various conditions, as described for panel (A). pRPA intensity was quantified using ImageJ software. The relative intensity of pRPA in the cells treated with shControl was arbitrarily set to 1. All data are expressed as mean ± SEM of at least three independent assays. *P*-values were calculated using two-tailed Student's *t*-tests. (**C**) Immunoprecipitation and western blot analysis of pRPA (s33) in HeLa cells under various conditions, as indicated in the panel.

A major function of hDNA2 in counteracting replication stress is resecting DNA ends at DSBs to generate 3′ single-stranded DNA (ssDNA) overhangs for homologous recombination during the repair of replication-related DSBs (13). To test if the 6KR hDNA2 mutation, which abolishes hTRAF6-mediated hDNA2 ubiquitination and nuclear localization, causes defects in ssDNA production during HDR, we expressed WT or 6KR in HeLa cells with hDNA2 stably knocked down (knockdown efficiency was confirmed by quantitative RT-PCR, Supplementary Figure S3A). We treated the HeLa cells with CPT (1 μM) to induced replication-associated DSBs (54). We used immunofluorescence and western blot analysis to evaluate the level of RPA phosphorylation (pRPA; S33), which is a common readout for ssDNAs arising during DNA repair (11). We observed that CPT induced a significant increase in pRPA levels in the cells (Figure 7). However, upon CPT treatment, pRPA levels were significantly lower in hDNA2 knockdown HeLa cells than in WT HeLa cells (Figure 7). Furthermore, CPT-induced pRPA levels were partially restored in hDNA2 knockdown HeLa cells upon expression of WT but not 6KR hDNA2 (Figure 7).

**Figure 8.**
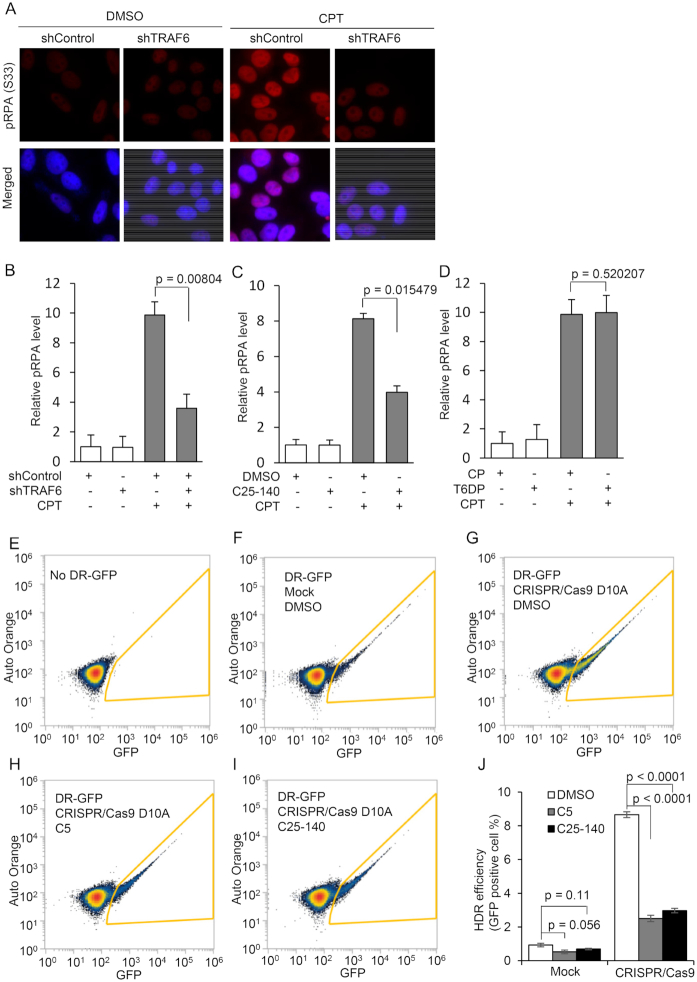
Inhibition of hTRAF6-mediated hDNA2 ubiquitination impairs DNA end resection and HDR (See also Supplementary Figures S3–5). (**A**–**D**) Immunofluorescence staining for pRPA (red) was conducted using an anti-pRPA (S33) antibody. Nuclei were stained with DAPI (blue). (A) Representative images of HeLa cells transfected with control shRNA (shControl) or shRNA against hTRAF6 (shTRAF6), followed by CPT treatment (1 μM, 4 h). (B) Relative pRPA levels in the control and TRAF6 knockdown cells treated with DMSO or CPT. (C) Relative pRPA levels in HeLa cells pretreated with DMSO or C25–140 (30 μM, 48 h) followed by treatment with DMSO or CPT (1 μM, 4 h). Representative images are shown in Supplementary Figure S4A. (D) Relative pRPA levels in HeLa cells that were pre-treated with a cell-permeable control peptide (CP) or T6PD peptide (100 μM, 24 h), followed by treatment with DMSO or CPT (1 μM, 4 h). Representative images are shown in Supplementary Figure S4B. pRPA intensity was quantified using ImageJ software. The relative pRPA intensity in the control cells (B: shControl/DMSO; C: DMSO/DMSO; D: CP/DMSO) was arbitrarily set to 1. All data are expressed as mean ± SEM of at least three independent assays. The *P*-values were calculated using two-tailed Student's *t*-tests. (**E**–**J**) CRISPR/Cas9 D10A-based DR-GFP HDR assay. 293T cells harboring the cassette for the DR-GFP reporter HDR assay were pretreated with DMSO (48 h), C5 (50 μM, 24 h) or C25–140 (30 μM, 48 h). The plasmid encoding CRISPR/Cas9 D10A nickase and a pair of sgRNAs was transfected into the 293T cells to induce DSB via double-nicking. HDR efficiency was analyzed using flow cytometry. (E–I) Representative flow cytometry plots for parental 293T cells or 293T DR-GFP cells under various conditions, as indicated in each panel. Counts within the yellow frame were scored as GFP-positive cells. (J) Percentages of GFP-positive cells among 293T DR-GFP cells under the conditions shown in panels (G–I). Data are expressed as mean ± SEM of at least three independent assays. The *P*-values were calculated using two-tailed Student's *t*-tests.

Given that hTRAF6-mediated hDNA2 ubiquitination promotes hDNA2 stability and nuclear localization, inhibition of this process is expected to cause defects in DNA end resection and HDR similar to those observed in 6KR mutant HeLa cells. To test this hypothesis, we knocked down hTRAF6 in HeLa cells (Supplementary Figure S3B). We measured pRPA levels using immunofluorescence staining in HeLa cells with or without hTRAF6 knockdown and treated with CPT. In the absence of CPT, pRPA levels were low and not significantly impacted by hTRAF6 knockdown (Figure 8A and B). CPT treatment induced an increase in pRPA levels, but this effect was inhibited by hTRAF6 knockdown (Figure 8A and B). Consistent with this finding, treatment with the TRAF6 E3 ligase activity inhibitor C25–140 similarly inhibited CPT-induced pRPA (Figure 8C and Supplementary Figure S4A). In contrast, treatment with the TRAF6/RANK interaction-inhibiting peptide T6DP had little effect on CPT-induced pRPA (Figure 8D and Supplementary Figure S4B). Next, we used a modified version of the CRISPR/Cas9-based direct repeat GFP (DR-GFP) reporter HDR assay (45) to test if hTRAF6 inhibition impairs overall HDR efficiency. In a modified version of the DR-GFP reporter assay (Supplementary Figure S5), we used the double-nicking CRISPR/Cas9 D10A system (55) rather than the WT CRISPR/Cas9 system, to specifically introduce DSBs at the indicated site to initiate HDR (Supplementary Figure S5). The CRISPR/Cas9 D10A-based DR-GFP construct was inserted into the 293T genome. Without the DR-GFP construct or the expression of sgRNA/Cas9 D10A nickase, using the multiplex CRISPR/Cas9 vector (46), <1% of GFP-positive cells were observed (Figure 8E, F and J). However, significantly more 293T cells were GFP-positive (Figure 8G and J, *P* < 0.0001) in the presence of DR-GFP and sgRNA/Cas9 D10A, which induced DSBs and subsequent HDR. However, the double-nicking-induced HDR of DR-GFP was significantly inhibited in cells pretreated with the DNA2 inhibitor C5 or the TRAF6 inhibitor C25–140 (Figure 8H–J; *P* < 0.0001 for both cases). These findings indicate that hTRAF6 inhibition suppresses HDR, which is consistent with our observations that hTRAF6 mediates hDNA2 nuclear translocation.

## Discussion

Biochemical and cellular analyses of the DNA2 molecule and studies of DNA2 knockout mice have provided convincing evidence for the essential roles of hDNA2 and other mammalian DNA2 proteins in efficient nuclear DNA replication and repair (11,13,27,31,37,56–58). However, the nuclease and helicase activities of DNA2 must be tightly controlled to avoid unwanted processing of DNA structures. This control may be achieved, at least in part, through a spatial regulation mechanism. Studies from our group and others have revealed that levels of nuclear hDNA2 are low under normal physiological conditions (36,37). In response to endogenous or environmental genotoxic stresses, however, hDNA2 migrates into the nucleus and subsequently binds to chromatin. What mediates the nuclear localization of human and other mammalian DNA2 molecules in the absence of an NLS remains unknown. The current study reveals that hTRAF6 catalyzes the K27- and K63-linked polyubiquitination of hDNA2 at its nuclease domain, which induces its nuclear localization. Polyubiquitination of hDNA2 may promote its binding to ubiquitin-binding domain-containing proteins that have an NLS. Thus, hDNA2 may be localized to the nucleus via co-transport with such proteins. Intriguingly, a recent study suggested that the K63-linked ubiquitin chain also has direct DNA-binding activity (59). Meanwhile, others have demonstrated that increased DNA-binding affinity is sufficient to drive NLS-independent nuclear localization of the nuclear protein MeCP2 (60). Therefore, it is also possible that the K63-linked ubiquitination of hDNA2 promotes its nuclear translocation by increasing its DNA-binding activity. In addition, a previous study showed that the monoubiquitination of the tumor suppressor pTEN enhances its stability and NLS-independent nuclear accumulation (41). The K63-linked ubiquitination of hDNA2 may similarly enhances its stability to passively increase its nuclear accumulation. On the other hand, we also notice that majority of 3xFlag-DNA2 in the nucleus with the molecular weight corresponding to the unmodified form. One possible explanation for this puzzling phenomenon is that hDNA2 polyubiquitination is a very dynamic process that is controlled by both the hTRAF6 E3 ligase and deubiquitinases. hTRAF6 catalyzes hDNA2 ubiquitination and drives its nuclear localization, and once in the nucleus, hDNA2 may be de-ubiquitinated. Consistent with this hypothesis, our unpublished studies indeed have revealed that hDNA2 interacts with the deubiquitinases USP24, USP39 and USP44.

The 6KR hDNA2 mutation, which abolishes hDNA2 ubiquitination, causes defects in DNA end resection and HDR similar to those observed in hDNA2 knockdown cells. hDNA2 has been shown to be important for DNA replication in difficult-to-replicate regions, such as centromeres and telomeres, and to play a role in restarting stalled replication forks (12,27,31,57). Because all of these pathways require the presence of hDNA2 in the nucleus, it is expected that the 6KR hDNA2 mutation will also result in centromere and telomere defects, contributing to late S/G2 cell-cycle arrest and genomic instability.

The current study also elucidates a new mechanism linking NF-κB signaling and the DNA damage response and repair pathways. The E3 ubiquitin ligase TRAF6 is a key component in the activation of NF-κB signaling by cytokines (61). IL1α and IL1β bind to their receptors to recruit the adaptor proteins MyD88, IRAK4 and IRAK1, leading to TRAF6 activation. Activated TRAF6 catalyzes the synthesis of unanchored K63-linked polyubiquitin chains, which bind to TAB2/TAK1 (61). The activated TAB2/TAK1 complex phosphorylates the IKK kinase complex to activate NF-κB (61). More recently, TRAF6 has been found to mediate the activation of NF-κB by DNA damage in an ATM-dependent manner (48). DNA damage, especially DSBs, due to genotoxic stresses is recognized by PARP1, which recruits and activates ATM (62). The activated ATM migrates to the cytoplasm and phosphorylates hTRAF6, which mediates K63 polyubiquitination to activate TAK1 and IKKs (48). Our current study extends this network, showing that hTRAF6 also catalyzes the polyubiquitination of hDNA2 to enhance hDNA2 stability and nuclear localization for DNA repair. These findings suggest that a key component in the NF-κB signaling pathway (TRAF6) is also used to transduce DNA damage response signals to regulate the nucleocytoplasmic trafficking of DNA repair proteins.

The importance of hTRAF6 in DNA repair is underscored by evidence that genetic or chemical inhibition of hTRFA6 impairs the function of hDNA2 in DNA end resection and consequently HDR, which is a major pathway for DSB repair and the rescue of stalled replication forks (63,64). Because TRAF6-mediated hDNA2 ubiquitination and nuclear localization occurs not only in response to stresses but under normal growth conditions, it is likely that these molecular mechanisms are also important in mediating other functions of hDNA2 in the nucleus, including centromere and telomere replication. These findings have important implications for the role of hTRAF6 in cancer initiation and progression. Indeed, hTRAF6 is overexpressed in human cancers and is association with cancer progression and poor prognosis (65–67). The role of hTRAF6 in promoting cancer is consistent with its role in the activation NF-κB, which induces the expression of many cell proliferation and survival genes (68). Furthermore, cancer cell survival and proliferation rely on the upregulation of DNA repair pathways to maintain the integrity of the nuclear genome in the face of stress due to aberrant DNA replication, which occurs at high levels in cancer cells (3,4). hDNA2, which we now show requires hTRAF6, is upregulated in response to replication stress and crucial for DNA repair processes (11,13,69). Thus, our current study suggests a new role for hTRAF6 in promoting cancer progression (3,4). Indeed, the inhibition of hTRAF6 not only blocks NF-κB-mediated cell survival signaling but also reduces the capacity of cancers cells to counteract DNA replication stress. Therefore, hTRAF6 may serve as an excellent target for cancer prevention and treatment. However, the genetic or chemical inhibition of hTRAF6 can impair the ability of DNA2 to maintain nuclear genome integrity under normal physiological conditions, which may contribute to cancer initiation. Therefore, it is important to be mindful that hTRAF6, like DNA repair genes, may have both tumor promoting and suppressive functions.

## Supplementary Material

gkz537_Supplemental_File
