# Supplementary material for: TRAF6 mediates human DNA2 polyubiquitination and nuclear localization to maintain nuclear genome integrity
Source: Nucleic Acids Res. 2019 Jun 19;47(14):7564–79. doi: 10.1093/nar/gkz537 (PMC6698806; doi:10.1093/nar/gkz537)
Supplement: gkz537_Supplemental_File [file gkz537_supplemental_file.pdf]

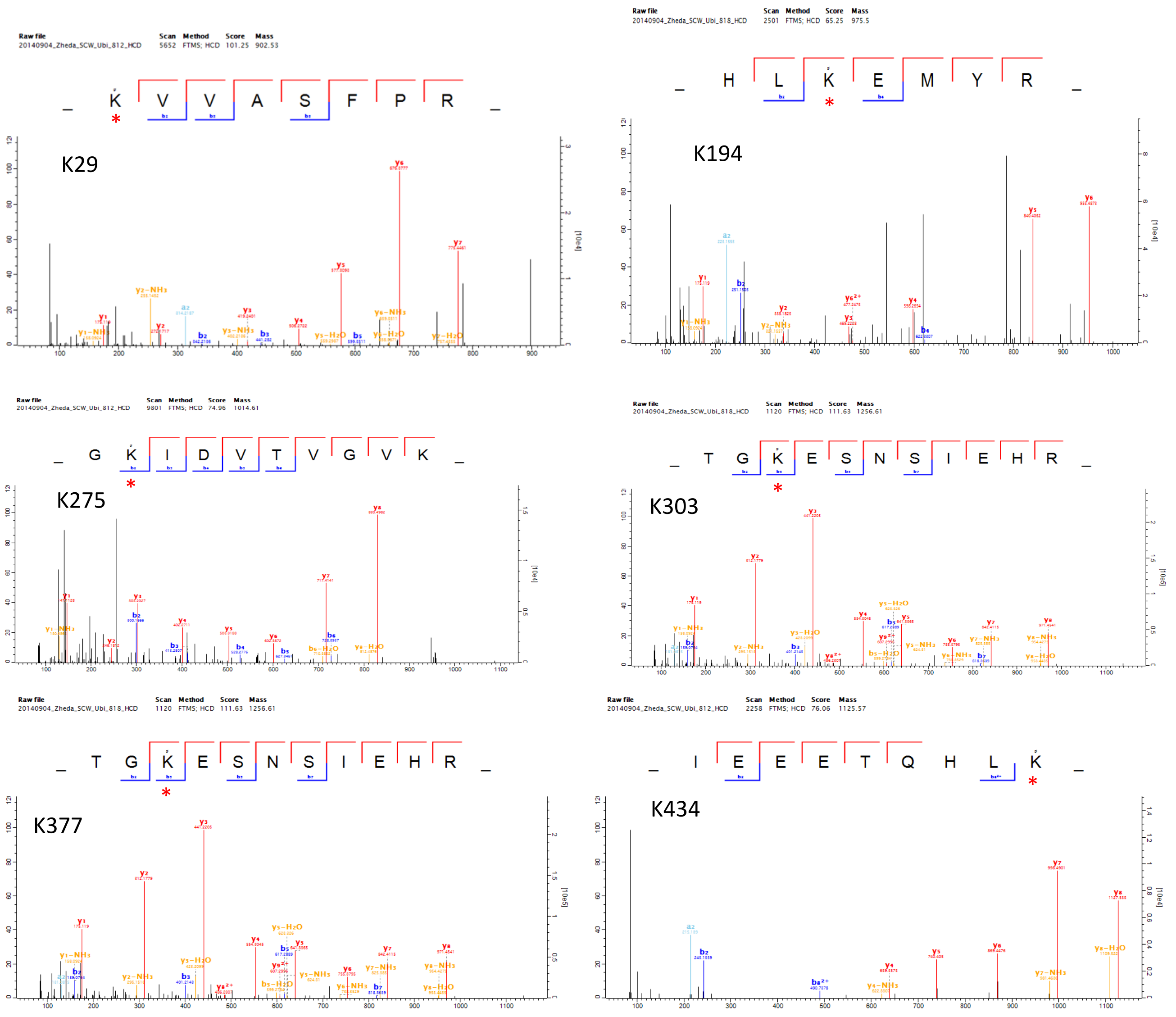

Figure S1. Identification of hDNA2 ubiquitination sites using liquid chromatography with tandem mass spectrometry (LC-MS/MS). 3xFlag-hDNA2 was isolated from 293T cells expressing 3xFlag-hDNA2, myc-hTRAF6, and 6His-ubiquitin; resolved by SDS-PAGE; and analyzed using LC-MS/MS. Ubiquitinated peptides were found using MaxQuant software. Mass spectrometry results for fragments of ubiquitinated K29, K194, K275, K303, K377, and K434 residues are shown. Asterisks (*) indicate ubiquitinated lysine residues. b and y ions are indicated in blue and red, respectively.

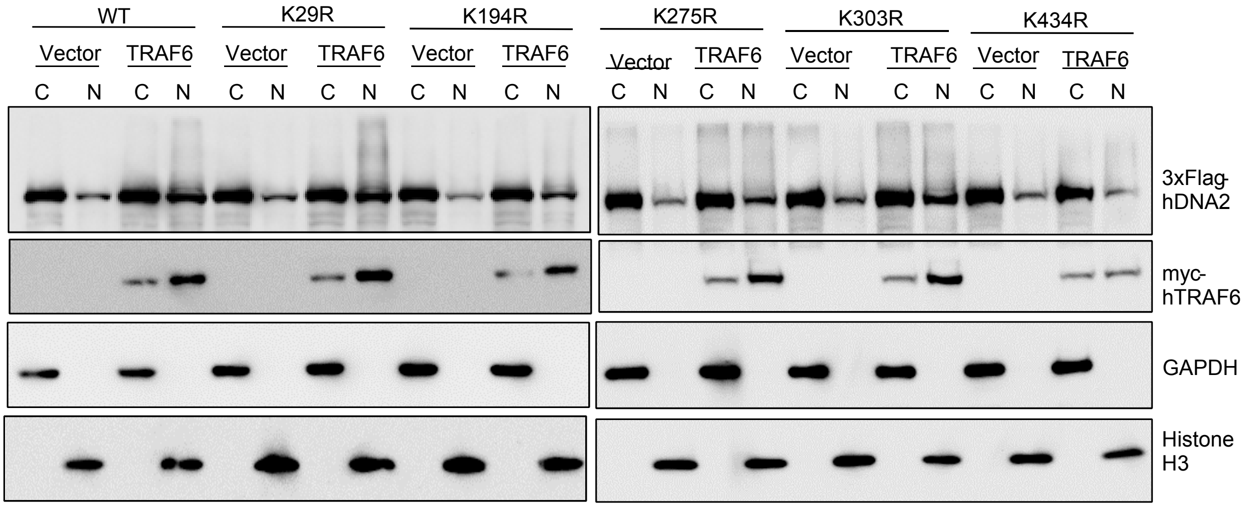

Figure S2. Nuclear localization of WT and hDNA2 mutants. Vectors encoding 3xFlag-tagged WT, K29R, K194R, K275R, K303R, or K434R hDNA2 were co-transfected with empty vectors or vectors encoding myc-hTRAF6 in 293T cells. 3xFlag-hDNA2 proteins in the CE and NE were analyzed by western blot analysis using an anti-Flag antibody. GAPDH and histone H3 were used as markers for the cytoplasmic and nuclear fractions, respectively.

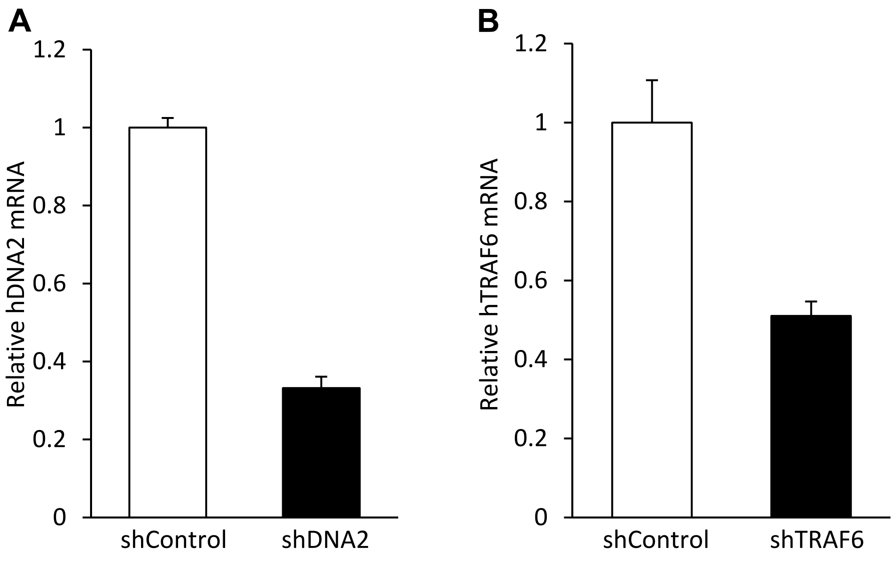

Figure S3. Confirmation of knockdown efficiency using quantitative RT-PCR. Relative levels of **(A)** hDNA2 and **(B)** hTRAF6 mRNA were measured in HeLa cells transfected with a control shRNA plasmid (shControl) or shRNA plasmids against hDNA2 or hTRAF6, respectively. Total RNA was extracted from these cells and quantitative RT-PCR was used to quantify the levels of hDNA2, hTRAF6, and control β-actin mRNA. hDNA2 and hTRAF6 mRNA levels were normalized to β-actin mRNA levels, and the normalized hDNA2 and hTRAF6 mRNA levels in shControl cells was arbitrarily set to 1. The values shown are the means ± s.e.m. of three independent assays.

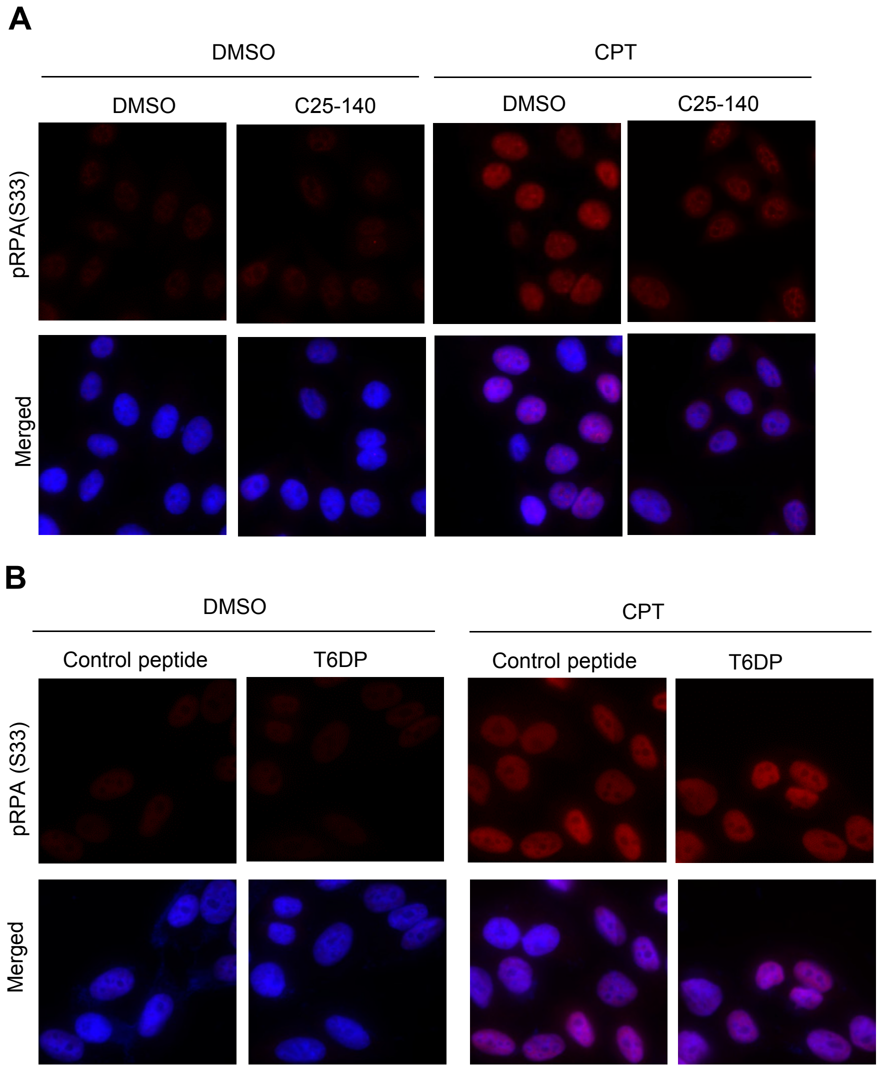

Figure S4. Representative immunofluorescence (IF) staining for pRPA (S33). HeLa cells were pretreated with **(A)** DMSO or C25-140 (30 μM, 48 h) or **(B)** a control or T6PD peptide (100 μM, 24 h). The cells were then treated with DMSO or CPT (1 μM, 4 h). In each case, red: pRPA (S33), blue: DAPI-stained nuclei.

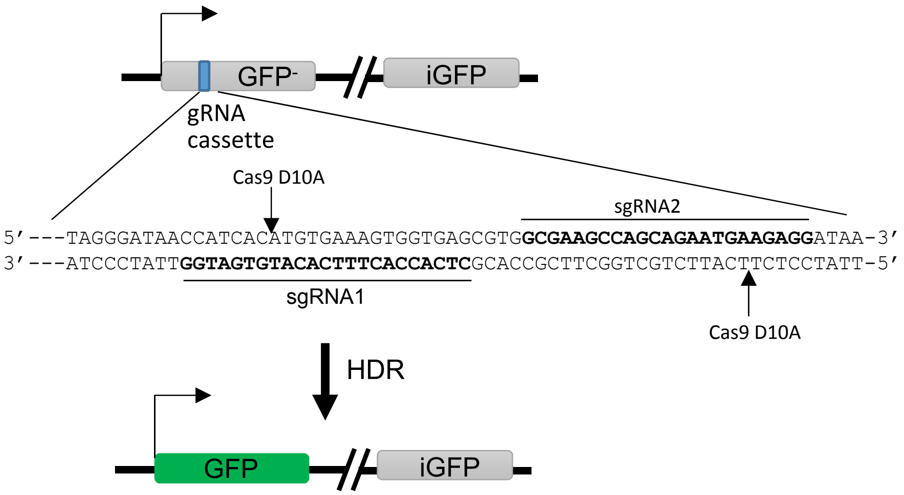

Figure S5. Schematic of the CRISPR/Cas9 D10A double-nicking-based DR-GFP reporter HDR assay. A cassette containing two CRISPR/CAS9 target sites (underlined) was inserted into the GFP gene (GFP-). Expression of Cas9 D10A nickase and a pair of single strand guide sequences (sgRNA1 and sgRNA2) results in a DSB. DNA end resection and subsequent recombination with a downstream GFP fragment (iGFP) will produce a functional GFP gene whose product can be detected and quantified using flow cytometry.
